# Evidence for the tonic inhibition of spinal pain by nicotinic cholinergic transmission through primary afferents

**DOI:** 10.1186/1744-8069-3-41

**Published:** 2007-12-19

**Authors:** Misaki Matsumoto, Weijiao Xie, Makoto Inoue, Hiroshi Ueda

**Affiliations:** 1 Division of Molecular Pharmacology and Neuroscience, Nagasaki University Graduate School of Biomedical Sciences, 1-14 Bunkyo-machi, Nagasaki 852-8521, Japan

## Abstract

**Background:**

We have proposed that nerve injury-specific loss of spinal tonic cholinergic inhibition may play a role in the analgesic effects of nicotinic acetylcholine receptor (nAChR) agonists on neuropathic pain. However, the tonic cholinergic inhibition of pain remains to be well characterized.

**Results:**

Here, we show that choline acetyltransferase (ChAT) signals were localized not only in outer dorsal horn fibers (lamina I–III) and motor neurons in the spinal cord, but also in the vast majority of neurons in the dorsal root ganglion (DRG). When mice were treated with an antisense oligodeoxynucleotide (AS-ODN) against ChAT, which decreased ChAT signals in the dorsal horn and DRG, but not in motor neurons, they showed a significant decrease in nociceptive thresholds in paw pressure and thermal paw withdrawal tests. Furthermore, in a novel electrical stimulation-induced paw withdrawal (EPW) test, the thresholds for stimulation through C-, Aδ- and Aβ-fibers were all decreased by AS-ODN-pretreatments. The administration of nicotine (10 nmol i.t.) induced a recovery of the nociceptive thresholds, decreased by the AS-ODN, in the mechanical, thermal and EPW tests. However, nicotine had no effects in control mice or treated with a mismatch scramble (MS)-ODN in all of these nociception tests.

**Conclusion:**

These findings suggest that primary afferent cholinergic neurons produce tonic inhibition of spinal pain through nAChR activation, and that intrathecal administration of nicotine rescues the loss of tonic cholinergic inhibition.

## Background

A potential analgesic effect of nicotine was claimed as early as the 1930s [1]. Since then, many researchers have demonstrated analgesic effects of nicotine and nicotinic acetylcholine receptor (nAChR) agonists such as epibatidine and ABT-594 [2,3]. Marubio et al. [4] also reported that the alpha4 nAChR subunit is crucial for nicotine-elicited analgesia. Nicotinic agonists are effective by systemic and intracerebroventricular administration [3,5-7], and intrathecal administration [7-10]. Recently, we demonstrated that these nicotinic agonists induced potent analgesia in mice with neuropathic pain using doses 5 or 10 times lower than those required in naïve mice [11]. Further pharmacological and electrophysiological findings suggest that this neuropathic pain-specific analgesia is related to a loss of tonic nicotinic stimulation to inhibitory GABAergic and glycinergic interneurons [11,12], in accordance with previous reports that nAChR agonists enhanced inhibitory GABAergic and glycinergic activities in the dorsal horn of the spinal cord [13-15].

However, little is known of the presence of acetylcholine (ACh) neurons in the spinal dorsal horn. There have been reports that dorsal root ganglion (DRG) neurons express several marker molecules for cholinergic neurons, including cholinergic vesicular acetylcholine transporter (VAChT) and choline acetyltransferase (ChAT) [16-18]. In addition, there ACh release from embryonic DRG explants has been shown [19]. On the other hand, there are many reports that neural injury reduces the biosynthesis of ACh in terms of ChAT expression, ChAT activity, ACh content and choline uptake [20-24]. Thus, it is interesting to speculate that primary afferent cholinergic neurons are responsible for the tonic inhibition of spinal pain.

In the present study we attempted to clarify the role of primary afferent neurons in this tonic inhibition and to reproduce nicotinic analgesia with low doses by down-regulation of ChAT activity, based on this speculation.

## Results

### ChAT expression in the spinal cord and DRG

**Figure 1:**
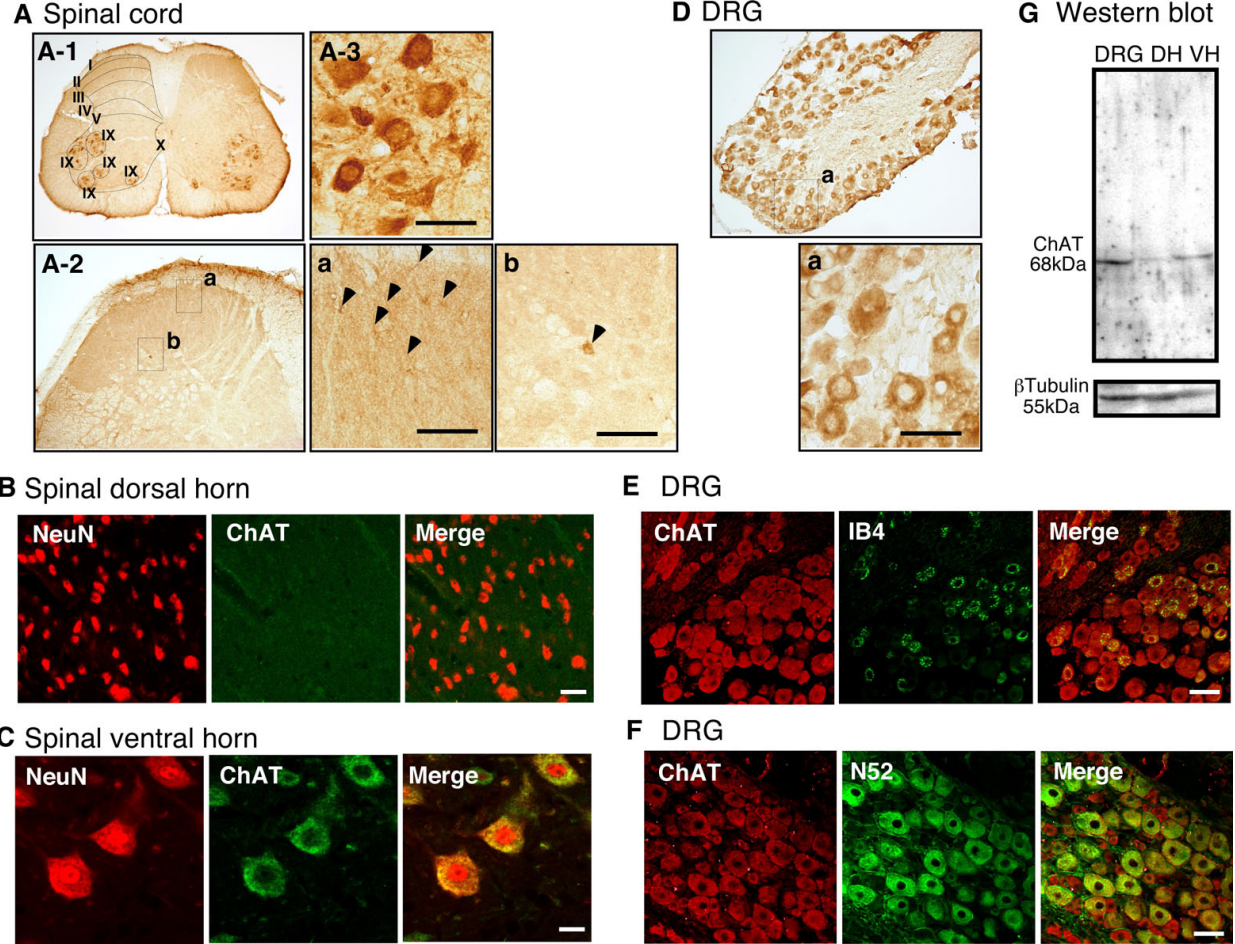
Choline acetyltransferase (ChAT) immunoreactivities in the spinal cord and DRG. (A) ChAT-immunoreactivities in fiber-like structures of lamina I–III regions of the dorsal horn, lamina X and in discrete lamina IX regions of ventral horn. Representative picture of fiber-like structures; discrete lamina IX regions are marked in the figure (A-1). (A-2) Many fiber-like structures (inset in a) were observed, in contrast to the very few ChAT-positive cell bodies (inset in b) observed, in dorsal laminae I–III. Representative picture of the intense ChAT signals found in the motor neurons of the lamina IX region (A-3). (B, C) Double-immunostaining for ChAT (green) and the neuronal marker NeuN (red) in the lamina I–III region (B), and in the lamina IX region (C). Note that ChAT-signals in the lamina I–III region (B) are not observed in neuronal cell bodies labeled by anti-NeuN IgG, while those in the lamina IX region (C) are always labeled by this IgG. (D) Representative picture of ChAT-immunoreactivities in the DRG. ChAT signals were found in most cells across the size spectrum (inset in a). (E) Double-immunostaining for ChAT (red) and the unmyelinated C-fiber marker IB4 (green) in the DRG. (F) Double-immunostaining for ChAT (red) and the myelinated A-fiber marker N52 (green). Scale bar = 20 μm for (B, C), and 50 μm for (A-2a, A-2b, A-3, D, E, F). (G) Western blot analysis using rabbit antiserum showing one 68-kDa immunoreactive band for ChAT. The 55-kDa immunoreactive band for β-tubulin is also indicated at the bottom. DH: spinal dorsal horn and VH: spinal ventral horn.

Using ChAT-specific rabbit antiserum [25,26], we performed immunohistochemical analysis of L4-5 spinal cords and DRG sections. Intense ChAT immunoreactivity was observed in the lamina IX regions of the ventral horn and the lamina X regions encircling the central canal, and modest activity was seen in fiber-like structures in the lamina I–III regions of the dorsal horn (Fig. 1A). Very few ChAT-positive cell bodies were observed in dorsal laminae I–III (Fig. 1A). From double-staining experiments using anti-NeuN and anti-ChAT antibodies, it was revealed that most immunoreactivities in the dorsal horn originated from fibers (Fig. 1B), while those in the ventral horn originated from large motor neurons (Fig. 1C). On the other hand, most cells in the DRG, from small to large, were ChAT-positive (Fig. 1D). All IB4-positive cells, indicated as unmyelinated C-fiber neurons, and N52-positive myelinated A-fiber neurons, in the DRG, were also ChAT-positive (Figs. 1E, 1F). No ChAT-activity was observed without ChAT antiserum (data not shown). Quite similar cytochemical results were also observed using a commercially available antibody from a different source (goat anti-ChAT polyclonal antibody, AB144P, Chemicon, CA) (see additional file 1A, B). Western blotting revealed only one 68-kDa immunoreactive band in protein lysates of DRGs, spinal dorsal horn and spinal ventral horn (Fig. 1G, and additional file 1C), indicating the high specificity of the rabbit antiserum and goat polyclonal antibody.

### Knock-down of ChAT in the DRG and spinal dorsal horn

**Figure 2:**
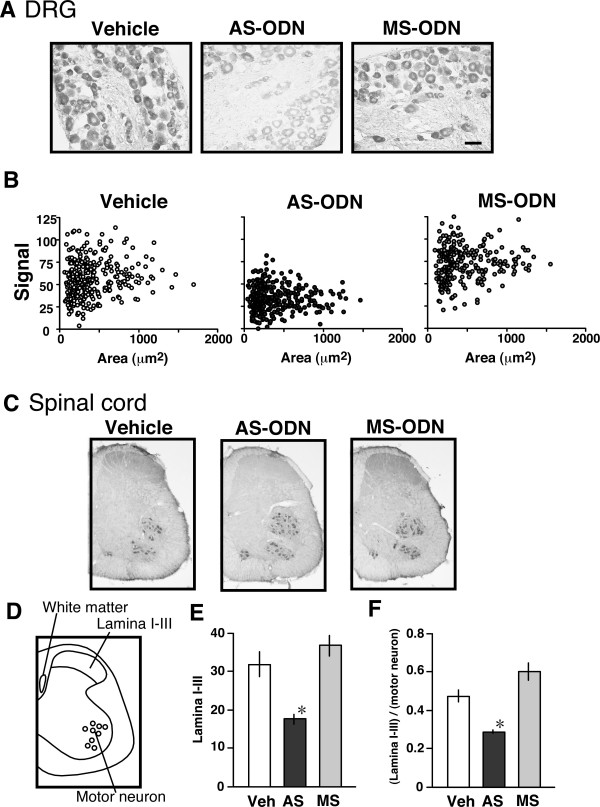
ChAT knock-down using an antisense oligodeoxynucleotide (AS-ODN) against ChAT, and decreased expression of ChAT in the DRG and spinal cord. (A, B) Down-regulation of ChAT activities by the AS-ODN in a DRG cross-section. Representative immunohistochemistry (A) and scatter diagram of ChAT activities (B) showing down-regulation of ChAT protein by the AS-ODN, but not by the MS-ODN. Immunoreactivity (B) was measured as an arbitrary value by automatic fluorescence microscopy using BZ Image Measurement software (Bio-Zero, Keyence, Tokyo, Japan). The evidence for down-regulation was reproduced in another experiment. (C) Representative pictures of ChAT-immunohistochemistry in the spinal cord treated with AS-ODN or MS-ODN. (D) Schematic diagram showing the region of interest. (E) Down-regulation of ChAT immunoreactivity by AS-ODN in laminae I–III of the dorsal horn. Each data point (average from 8 sections) was calculated by the formula [(signal/area in lamina I–III) - (signal/area in gracile fasciculus regions of white matter)]. Results represent the means ± S.E.M. from 3 separate mice. *p < 0.05 vs. vehicle. (F) Selective down-regulation of ChAT immunoreactivity by the AS-ODN in laminae I–III of the dorsal horn. Each point of data (average from 8 sections) was calculated by the formula [(signal/area in lamina I–III) - (signal/area in gracile fasciculus regions of white matter)]/[(signal/area in motor neurons) - (signal/area in white matter)]. Veh: vehicle, AS: AS-ODN and MS: MS-ODN. Results represent the means ± S.E.M. from 3 separate mice. *:*p *< 0.05 vs. vehicle. Scale bar = 50 μm for (A).

Mice were intrathecally treated with an antisense oligodeoxynucleotide (AS-ODN) to ChAT or the corresponding mismatch scrambled (MS)-ODN on the 1st, 3rd and 5th days of the experiment, and perfused transcardially with 4% PFA solution on the 6th day. DRGs and spinal cord at the L4-5 level were then isolated. As shown in Figs. 2A and 2B, ChAT-positive immunoreactivities in most cells were reduced by AS-ODN treatments, but not by MS-ODN treatments. Fiber-like immunoreactivities in the dorsal horn were reduced by AS-ODN, but not by MS-ODN, but the immunoreactivities in motor neurons were not affected (Fig. 2C). In order to quantify the change in the intensity of ChAT-immunoreactivity in the dorsal horn lamina I–III, we evaluated the intensities in the gracile fasciculus regions of white matter (as a background), the lamina I–III layer and motor neurons in the ventral horn (Fig. 2D). As shown in Figs. 2E and 2F, either ChAT-immunoreactivities in laminae I–III or the ratio of the level in the lamina I–III layer to that in motor neurons was significantly reduced by the AS-ODN, but not by the MS-ODN. However, there was no reduction in ChAT level in the ventral motor neurons due to the AS-ODN (data not shown).

### Reduced nociceptive thresholds by ChAT-knock down

**Figure 3:**
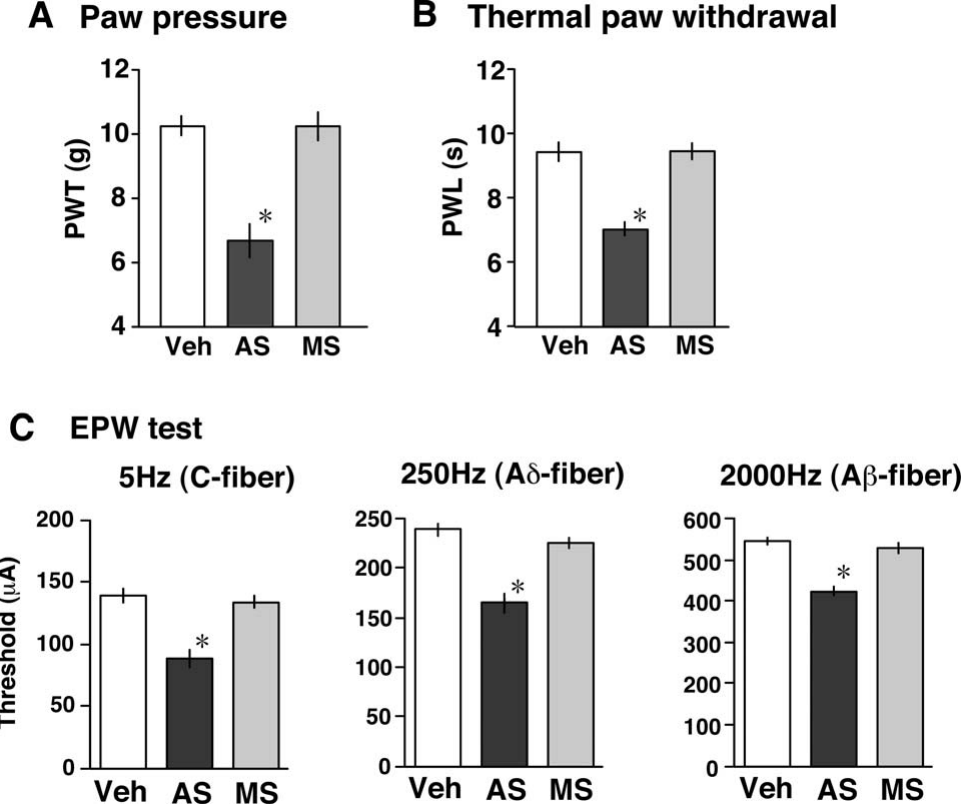
Reduction in the nociceptive threshold in various tests following pretreatment with an AS-ODN against ChAT. The threshold represents the weight (paw withdrawal threshold: PWT, in g) required to induce withdrawal behavior in the paw pressure test (A), latency (paw withdrawal latency: PWL, in s) in the thermal paw withdrawal test (B) and minimum intensity (μA) in the EPW test using 5, 250 and 2000 Hz electrical stimuli. Tests of nociception were performed on the 6th day after the start of pretreatments with vehicle (Veh), AS-ODN (AS) or MS-ODN (MS). *p < 0.05 *vs. *vehicle. Data represent the means ± S.E.M. from experiments using at least 6 mice.

Nociception tests were performed on the 6th day after the start of pretreatments with AS-ODN or MS-ODN. In the paw pressure mechanical test and the thermal paw withdrawal test, the average ± S.E.M. vehicle control thresholds were 10.21 ± 0.29 g (n = 6) and 9.36 ± 0.30 s (n = 6), respectively. AS-ODN pretreatment significantly reduced these thresholds, while MS-ODN pretreatment did not, as shown in Fig. 3A and 3B.

We recently established an electrical stimulation-induced paw withdrawal (EPW) test, which distinguishes between responses mediated by different sensory fibers [27]. In this novel nociception test, the foot of the hind limb was given transcutaneous nerve stimuli with sine-wave pulses of different frequencies of 5, 250 or 2000 Hz, to activate C-, Aδ- or Aβ-fibers, respectively [28,29], and the intensity (μA) inducing a withdrawal reflex was defined as the threshold. The average ± S.E.M. vehicle control thresholds for 5, 250 and 2000 Hz stimuli were 138.0 ± 5.3, 237.0 ± 6.7 and 541.0 ± 9.8 μA, respectively. AS-ODN pretreatments significantly reduced the thresholds at all three frequencies of stimuli to a similar degree, as shown in Fig. 3C (5 Hz, 87.2 ± 6.9 μA; 250 Hz, 162.7 ± 9.0 μA; 2000 Hz, 418.2 ± 10.4 μA). However, MS-ODN pretreatments induced no change at all.

### Nicotine rescues the reduced nociceptive thresholds by ChAT knock-down

**Figure 4:**
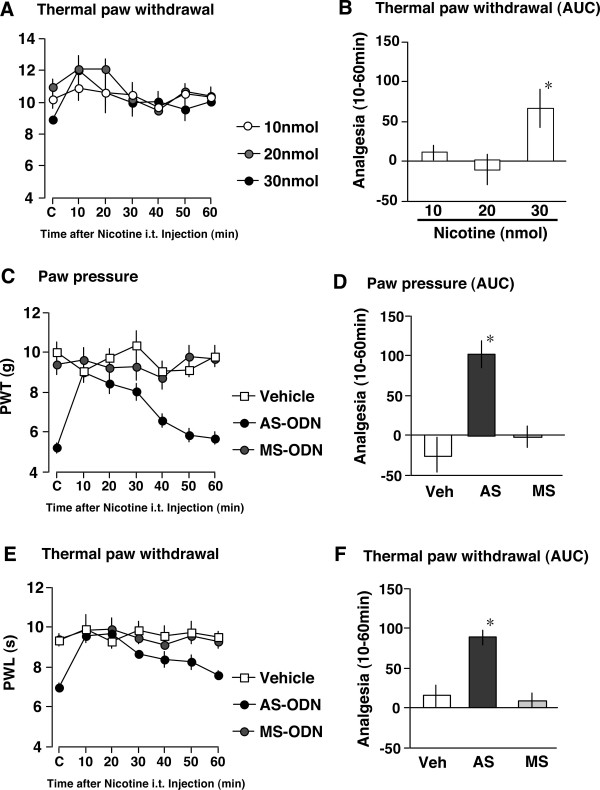
Specific analgesic effects of nicotine in ChAT AS-ODN-pretreated mice in paw pressure and thermal paw withdrawal tests. (A, B) Thermal paw withdrawal test in naïve mice. Time-course of paw withdrawal latency (s) after nicotine (10 nmol, 20 nmol and 30 nmol i.t.) injection (A). Comparison of analgesia (in AUC) following nicotine injection (B). (C, D) Paw pressure test. Time-course of paw withdrawal threshold (g) after nicotine (10 nmol i.t.) injection (C). Comparison of analgesia (in AUC) following nicotine injection (D). (C, D) Thermal paw withdrawal test. Time-course of paw withdrawal latency (s) after nicotine (10 nmol i.t.) injection (E). Comparison of analgesia (in AUC) following nicotine injection (F). *p < 0.05 vs. vehicle. Data represent the means ± S.E.M. from experiments using at least 6 mice.

**Figure 5:**
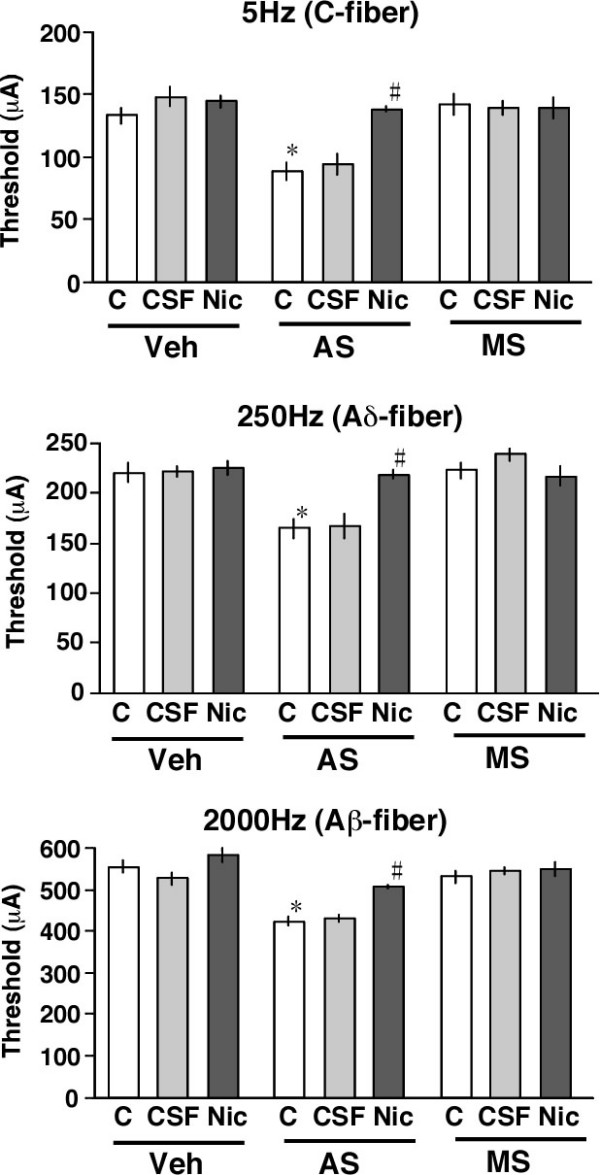
Specific analgesic effects of nicotine in ChAT AS-ODN-pretreated mice in the EPW test. The EPW test was performed 10 min after nicotine injection. Veh: vehicle, AS: AS-ODN, MS: MS-ODN, C: control, CSF: aCSF and Nic: nicotine. *p < 0.05 vs. vehicle. #*p *< 0.05 vs. aCSF. Data represent the means ± S.E.M. from experiments using at least 6 mice.

In naïve mice, 30 nmol of nicotine (i.t.) produced an analgesic effect in a thermal nociception test, but 10 or 20 nmol of nicotine did not (Figs. 4A, 4B). However, as mice given 30 nmol of nicotine showed side effects including hypolocomotion and nocifensive behavior (data not shown), this analgesic effect might be artifactual. In this study, we chose 10 nmol of nicotine (i.t.), which showed an analgesic effect against the hyperalgesia in animals with neuropathic pain, without any side effects [11]. In a paw pressure test using vehicle-treated mice, i.t. injection of nicotine had no effect on the nociceptive threshold throughout experiments, for 60 min (Fig. 4C). As mentioned above, AS-ODN pretreatment significantly reduced the threshold for thermal nociception on the 6th day after the start of treatments (Fig. 4C). Nicotine administration completely reversed this reduction in nociceptive threshold induced by AS-ODN-pretreatments, at the 10 min time point. The nicotine analgesia lasted for approximately 60 min, and quantitative analysis using area under the curve (AUC) analysis also showed similar results (Fig. 4D). However, neither hyperalgesia nor nicotine analgesia was observed in MS-ODN-pretreated mice (Fig. 4C, 4D). Similar results were also observed in the thermal pain test (Figs. 4E, 4F). Furthermore, nicotine-induced analgesia was also observed specifically in AS-ODN-pretreated mice, which showed reduced thresholds for nociception of 5, 250 or 2000 Hz stimuli (Fig. 5).

## Discussion

We found a number of fiber-like ChAT signals in the spinal dorsal horn, consistent with previous studies [30,31]. As these fiber-like signals were characteristically localized in the lamina I–III layers, it is suggested that such signals are principally derived from primary afferent fibers. This view is supported by the present study, showing that an AS-ODN against ChAT caused selective down-regulation of ChAT protein in both the DRG and the spinal dorsal horn, but not in motor neurons in the spinal ventral horn. Regarding this issue, we have observed that an intrathecally administered FITC-labeled AS-ODN is more efficiently distributed to the DRG than to the spinal cord, 30 min after administration [32]. This phenomenon may be explained on the possibility that acidic, large molecules are preferentially transported to the DRG, rather than the spinal cord. The present data demonstrate that AS-ODN pretreatment caused selective down-regulation of DRG-originating ChAT protein in the dorsal horn. The lack of a reduction of ChAT signals in the ventral horn can be explained by a lower permeability of the AS-ODN into the spinal cord.

ChAT signals were found in most cells in the DRG, including the IB4-positive unmyelinated and N52-positive myelinated neurons. Quite similar cytochemical results were also observed using a commercially available antibody from a different source (goat anti-ChAT polyclonal antibody; additional file 1). These data suggest that ChAT immunoreactivities in the spinal dorsal horn are derived from the fibers of myelinated and unmyelinated types of primary afferent cholinergic neurons. However, previous studies detected ChAT signals predominantly in small-diameter neurons of the rat DRG [16,17]. In addition, Bellier and Kimura [16] demonstrated that the small size of ChAT splice variant (55 kDa; pChAT) was only observed in the rat DRG. However, they failed to detect the large size of ChAT splice variant (68 kDa; cChAT). Our present study shows that small- and large-diameter DRG neurons in mice are ChAT positive. Western blot studies revealed that cChAT alone was detected in the mouse DRG with the ChAT-antiserum and with anti-ChAT antibody (AB144P, Chemicon, CA). Therefore the discrepancy in ChAT expression between the study by Bellier and Kimura and ours may be attributed to a species difference (rats *vs. *mice). Because the two antibodies used in our study do not detect pChAT, we cannot evaluate the DRG distribution of the pChAT variant. However, as we designed the AS-ODN against ChAT in a region common to both cChAT and pChAT (the region flanking the start codon ATG), it is expected to also down-regulate pChAT if present.

It is important to discuss the physiological role of primary afferent cholinergic innervation in terms of pain transmission. Previous studies have demonstrated the presence of cholinergic markers in the DRG [16-18], but the physiological role of ACh in these neurons remains to be determined. The present study firstly demonstrated the evidence for an inhibitory role for cholinergic primary afferents in spinal pain mechanisms. Pretreatments with a ChAT AS-ODN, which reduced the ChAT protein level in DRG neurons and in the terminal region of the spinal dorsal horn, led to significant decreases in the thresholds for mechanical and thermal nociception. These data are consistent with our previous data showing that intrathecal treatments with nAChR antagonists (mecamylamine) produce a reduction in the thresholds for mechanical and thermal nociception [12].

We have proposed that neuropathic pain-specific nicotine-induced analgesia is mediated by inhibitory GABA interneurons based on the findings that intrathecal treatment with a GABA receptor antagonist (picrotoxin) also produced a reduction in nociceptive thresholds, and the analgesic effect of nicotine was abolished by pretreatment with the GABA receptor antagonists bicuculline and picrotoxin [11]. The presence of tonic nAChR activation of inhibitory GABA interneurons is supported by many reports that nAChR agonists enhance inhibitory postsynaptic currents (IPSCs), possibly through GABAergic or glycinergic activities in the dorsal horn of the spinal cord [12-15,33]. Thus, the nicotine-induced analgesic effects in AS-ODN treated mice are likely to be attributed to the loss of tonic nAChR activation of inhibitory GABA interneurons. In other words, pain-inhibitory GABA interneurons are maximally or submaximally activated by tonic nicotinic stimulation. Alternatively, the nicotine-induced analgesia observed in the present study may be explained by supersensitivity of nAChR due to down-regulation of cholinergic activities by ChAT AS-ODN. However, the details of this possibility remain to be determined.

**Figure 6:**
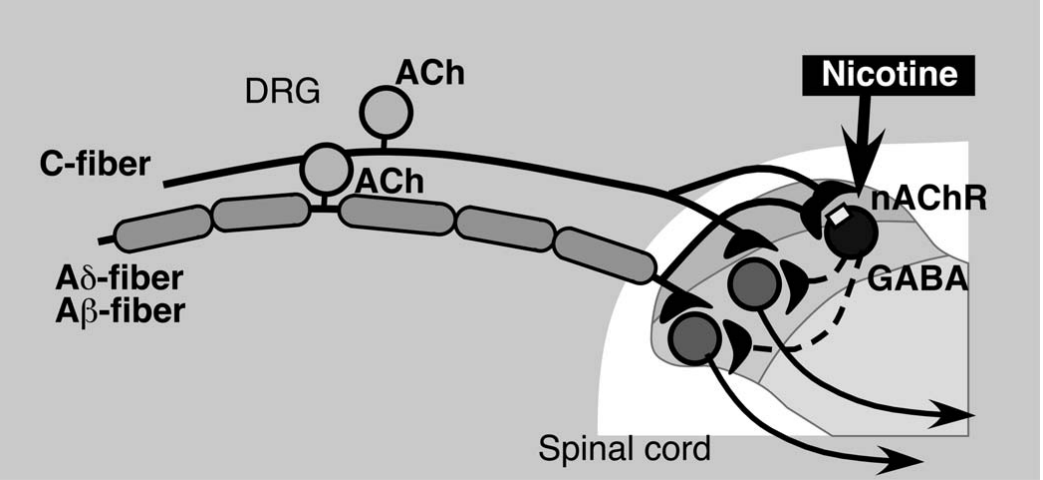
Working hypothesis for the tonic inhibition of spinal pain by nicotinic cholinergic transmission through primary afferents. The cholinergic primary afferents terminating in the spinal dorsal horn tonically activate nicotinic acetylcholine receptors on GABAergic interneurons through acetylcholine synthesis and release. Due to maximal activation of this network, exogenously administered nicotine has no effect in the normal state. On the other hand, in the ChAT knock-down state, the thresholds of pain mechanisms are reduced because of the loss of this tonic pain inhibition system, and exogenously applied nicotine can activate nAChRs leading to analgesia. The physiological role of ACh in DRG neurons is as a modulator of pain transmission to avoid hyperexcitability of spinal sensory neurons.

In this study, we attempted to examine the thresholds to stimuli through three different types of sensory fiber using the EPW test in control and ChAT AS-ODN-treated mice. As previously reported [34,35], the nociception of stimuli of different frequencies, specifically 5, 250 and 2000 Hz, was characterized to be through C, Aδ and Aβ-fibers, respectively. In the present study, ChAT AS-ODN-pretreatments decreased the thresholds to stimuli through all C-, Aδ- and Aβ-type fibers. These findings are consistent with the immunohistochemical data in the DRG: extensive distribution of ChAT protein was observed in most DRG neurons, including unmyelinated C- and myelinated A-fiber neurons. As it has been observed that ACh is released from DRG explants [19], we propose that primary afferent cholinergic innervation may inhibit all three types of sensory input through inhibitory GABA neurons, as illustrated in Fig. 6.

We have reported that nicotine shows potent analgesic effects, specifically in mice with sciatic nerve injury using doses at which these compounds had no effects in naïve mice [11]. To explain this result, we proposed that intrathecally administered nicotine rescues the loss of tonic cholinergic inhibition through GABA interneurons in neuropathic pain. The present experiments, which attempted to reproduce the loss of tonic cholinergic inhibition by pretreatment of animals with an AS-ODN against ChAT, may support this view. Recently, long-term potentiation (LTP) has been widely studied as a potential mechanism for central sensitization (spinal/cortical) in animals with neuropathic pain [36,37]. In this mechanism, LTP is facilitated by treatments with GABA antagonists [37]. Thus, the cholinergic innervation to GABA neurons may have a further influence on the LTP mechanism, in addition to tonic effects. The next step will be to examine whether or not the activity of the cholinergic primary afferent system is altered in a neuropathic pain model.

In the present study we demonstrated that primary afferent cholinergic neurons mediate tonic inhibition of spinal pain through nAChR, since intrathecal treatments with nicotine almost entirely normalized nociceptive thresholds that had been reduced by a ChAT AS-ODN. However, there are a number of reports that muscarinic receptors are also involved in the tonic inhibition of pain in the spinal cord [38,39]. Thus, it will be interesting to examine whether muscarinic agonists have similar potent analgesic effects in mice treated with a ChAT AS-ODN.

## Conclusion

This study demonstrates that primary afferent cholinergic neurons produce tonic inhibition of spinal pain through nAChR, and that intrathecal administration of nicotine rescues the loss of tonic cholinergic inhibition. This mechanism may be important for modulating spinal pain perception especially in pathological conditions, such as neuropathic pain.

## Methods

### Animals

Male ddY mice weighing 20–24 g were used after adaptation to the laboratory conditions: 22 ± 2°C, 55 ± 5% relative humidity and a 12 h light/dark cycle with food and water ad libitum. All procedures were approved by Nagasaki University Animal Care Committee and complied with the recommendations of the International Association for the Study of Pain [40].

### Tissue preparation

For immunohistochemistry, mice were deeply anesthetized with sodium pentobarbital (50 mg/kg i.p.) and perfused transcardially with 20 ml of potassium-free phosphate-buffered saline (K^+^-free PBS, pH 7.4), followed by 50 ml of a 4% paraformaldehyde (PFA) solution. The L4-5 DRGs and L4-5 spinal cord were isolated, postfixed for 3 h, and cryoprotected overnight in a 25% sucrose solution. Tissues were fast-frozen in cryo-embedding compound in a mixture of ethanol and dry ice and stored at -80°C until use. DRGs were cut on a cryostat at a thickness of 10 μm, thaw-mounted on silane-coated glass slides, and air-dried overnight at room temperature (RT). Spinal cords were cut on a cryostat at a thickness of 30 μm, collected in PBS solution containing 0.1% sodium azide, and processed as free-floating sections.

### Immunohistochemistry

DRG, but not spinal cord sections, were pretreated in 10 mM citrate buffer (pH 6.0) for antigen unmasking, using a microwave for 15 min, before immunolabeling. Slide-mounted DRG sections and free-floating spinal cord sections were incubated in 0.3% H_2_O_2 _in methanol for 10 min and washed with PBST (0.1% Triton X-100 in PBS). The sections were incubated with blocking buffer containing 1% BSA in PBST and subsequently reacted with rabbit antiserum against ChAT (1:3000 dilution in blocking buffer), which was kindly provided by Dr. H Misawa (Kyoritsu College of Pharmacology, Tokyo, Japan), overnight at 4°C [25,26]. After thorough washing, the sections were incubated with secondary antibody, biotinylated anti-rabbit IgG (1:500; Vector, CA), for 60 min at RT, and subsequently with ABC complex (Vector, CA) at RT for 60 min. ChAT-activities were visualized by incubation with a solution containing 0.02% 3,3'-diaminobenzidine tetrahydrochloride (DAB; Dojindo, Japan) and 0.0051% H_2_O_2 _in 0.05 M Tris-HCl buffer (pH 7.6), until brown reaction products appeared. Sections were mounted on glass slides, air-dried, dehydrated through a series of ethanol solutions, cleared in xylene, and coverslipped with PermaFluor (Thermo Shandon, Pittsburgh, PA). For double immunolabeling, we used the following antibodies: a mouse monoclonal antibody against the N52 clone of neurofilament 200, a marker of myelinated fibers (anti-N52; 1:30000; Sigma, St. Louis, MO); a mouse monoclonal antibody against neuron-specific nuclear protein (anti-NeuN; Chemicon, CA); biotin-conjugated BSI-B4 (10 μg/mL; Sigma, St. Louis, MO); Alexa Fluor 488-conjugated anti-mouse IgG; Alexa Fluor 594-conjugated anti-mouse IgG; Alexa Fluor 488-conjugated anti-rabbit IgG; Alexa Fluor 594-conjugated anti-rabbit IgG and Alexa Fluor 488-conjugated streptavidin (1:300; Molecular Probes, CA). The signal intensity of ChAT-immunoreactivity and the cross-sectional areas of DRG neurons were measured by automatic fluorescence microscopy using BZ Image Measurement software (Bio-Zero, Keyence, Tokyo, Japan). The sections shown in additional file 1 were incubated with a goat polyclonal antibody against ChAT (1:100; AB144P, Chemicon, CA), and subsequently reacted with biotinylated anti-goat IgG (1:500; Vector, CA).

### Western blotting

L4-6 DRGs, L4-5 spinal dorsal horn and spinal ventral horn proteins (40 μg) were separated on SDS-polyacrylamide gels (8%). Rabbit antiserum against ChAT (Fig. 1G) or goat polyclonal antibody against ChAT (additional file 1) was used at a dilution of 1:3000 or 1:500, respectively. HRP-labeled anti-rabbit antibody or HRP-labeled anti-goat antibody was used as a secondary antibody at a dilution of 1:1000. Visualization of immunoreactive bands was performed by the Light Capture (AE-6960/C/FC, Atto, Tokyo, Japan) with an enhanced chemiluminescent substrate for the detection of horseradish peroxidase, SuperSignal^® ^West Pico Chemiluminescent Substrate (PIERCE, Rockford, IL).

### Oligonucleotide treatments

The antisense oligodeoxynucleotide against ChAT (GenBank accession number: NM_009891) (AS-ODN; 5'-GGA TAG GCA TCC TAG CGA TT-3') and its mismatch scrambled oligodeoxynucleotide (MS-ODN; 5'-GAG TGA GCA CTC TAG CAG TT-3') were synthesized (Operon Biotechnologies, Tokyo, Japan), freshly dissolved in artificial CSF (aCSF) comprising 125 mM NaCl, 3.8 mM KCl, 2.0 mM CaCl_2_, 1.0 mM MgCl_2_, 1.2 mM KH_2_PO_4_, 26 mM NaHCO_3 _and 10 mM D-glucose (pH 7.4) and injected intrathecally (i.t.) between the L5 and L6 lumbar space in unanesthetized mice using a 30-gauge needle. This treatment was performed (10 μg/5 μl) on the 1st, 3rd and 5th days of the experiment, as stated in our previous study [41]. On the 6th day, treated mice were assessed in behavioral tests, and tissues were isolated for immunohistochemical experiments.

### Nociception tests

In thermal paw withdrawal tests, the nociception threshold was evaluated by the latency to paw withdrawal upon a thermal stimulus [42-44]. Unanesthetized animals were placed in plexiglas cages on top of a glass sheet and an adaptation period of 1 h was allowed. The thermal stimulator (IITC Inc., Woodland Hills, CA, USA) was positioned under the glass sheet and the focus of the projection bulb was aimed exactly at the middle of the plantar surface of the animal. A mirror attached to the stimulator permitted visualization of the plantar surface. A cut-off time of 20 s was set in order to prevent tissue damage. The mechanical paw pressure test was performed as described previously [43,44]. Briefly, mice were placed in a plexiglass chamber on a 6 × 6 mm wire mesh grid floor and were allowed to acclimatize for a period of 1 h. The mechanical stimulus was then delivered to the middle of the plantar surface of the right-hind paw using a Transducer Indicator (Model 1601, IITC Inc., Woodland Hills, USA). The pressure needed to induce a flexor response was defined as the pain threshold. In these experiments using mechanical and thermal tests, the thresholds were determined by three repeated challenges at 10 min intervals, and the averages of responses were evaluated. For the time-course experiment, we measured the threshold every 10 min only once, for a period of 1 h after i.t. injection. In the "area under the curve" (AUC) analysis of the nicotine effect on pain thresholds, we calculated the area under the curve generated by plotting analgesic threshold (by deducting the control threshold from each threshold point) against time, from 10 to 60 min after nicotine treatment, using a trapezoidal method. An electrical stimulation-induced paw withdrawal (EPW) test was performed as described previously [27]. Briefly, electrodes (Neurotron Inc., Blatimore, MD) were fastened to the right plantar surfaces and insteps of mice. Transcutaneous nerve stimuli consisting of three sine-wave pulses (5, 250 and 2000 Hz) were applied using a Neurometer CPT/C (Neurotron Inc.). The minimum intensity (μA) at which each mouse withdrew its paw was defined as the current stimulus threshold. Investigators blinded to drug-treatments performed all behavioral experiments.

### Nicotine treatment

Freebase (-)nicotine (Wako Pure Chemical Industries, Osaka, Japan) was freshly dissolved in artificial cerebrospinal fluid (aCSF), and injected intrathecally (i.t.) between the L5 and L6 lumbar space in unanesthetized mice using a 30-gauge needle, as stated in our previous study [11]. The behavioral threshold was measured at 10 min after i.t. injection. For the time-course experiment, we measured the threshold every 10 min for 1 h after i.t. injection.

### Statistical analysis

Differences between multiple groups were analyzed using a one-way ANOVA with Tukey-Kramer multiple comparison post-hoc analysis. Changes in the thresholds in the EPW test, with and without nicotine treatment, were analyzed using an unpaired Student's *t*-test. The criterion of significance was set at *p *< 0.05. All results are expressed as means ± S.E.M.

## List of abbreviations

nAChR: Nicotinic acetylcholine receptor;

ChAT: Choline acetyltransferase; 

AS-ODN: Antisense oligodeoxynucleotide; 

MS-ODN: Mismatch scrambled oligodeoxynucleotide; 

EPW: Electrical stimulation-induced paw withdrawal; 

DRG: Dorsal root ganglion.

## Competing interests

The author(s) declare that they have no competing interests.

## Authors' contributions

MM is responsible for experimental design, performance of behavioral and immunohistochemical analysis, and writing the manuscript. WX participated in behavioral experiments. MI participated in designing the study. HU supervised the experiments and writing the manuscript. All authors read and approved the final manuscript.

## Supplementary Material

Additional file 1 ChAT-immunoreactivities in the spinal cord and DRG, using a commercially available antibody from a different source (goat anti-ChAT polyclonal antibody, AB144P, Chemicon, CA). (A) ChAT-immunoreactivities in whole spinal cord. (A-1) Representative picture of ChAT-immunohistochemistry in the spinal cord. Many fiber-like structures in the dorsal horn (A-2) and intense ChAT-like signals in the lamina IX region (A-3) were observed. (B) ChAT-immunoreactivities in the DRG. (B-1) Representative pictures of ChAT-immunohistochemistry in the DRG. ChAT signals were found in most cells across the size spectrum (B-2). Scale bar = 50 μm for (A-2, A-3, B-2). (C) Western blot analysis using goat polyclonal antibody indicates one 68-kDa immunoreactive band for ChAT. The 55-kDa immunoreactive band for β-tubulin is also indicated at the bottom. DH: spinal dorsal horn and VH: spinal ventral horn. Click here for file

## References

[B1] Davis L, Pollock LJ, Stone TT (1932). Visceral pain. Surg Gynecol Obstet.

[B2] Spande TF, Garraffo HM, Edwards MW, Yeh HJC, Pannell L, Daly JW (1992). Epibatidine: a novel (chloropyridyl)azabicycloheptane with potent analgesic activity from an Ecuadoran poison frog.. J Am Chem Soc.

[B3] Bannon AW, Decker MW, Holladay MW, Curzon P, Donnelly-Roberts D, Puttfarcken PS, Bitner RS, Diaz A, Dickenson AH, Porsolt RD, Williams M, Arneric SP (1998). Broad-spectrum, non-opioid analgesic activity by selective modulation of neuronal nicotinic acetylcholine receptors. Science.

[B4] Marubio LM, del Mar Arroyo-Jimenez M, Cordero-Erausquin M, Lena C, Le Novere N, de Kerchove d'Exaerde A, Huchet M, Damaj MI, Changeux JP (1999). Reduced antinociception in mice lacking neuronal nicotinic receptor subunits. Nature.

[B5] Sahley TL, Berntson GG (1979). Antinociceptive effects of central and systemic administrations of nicotine in the rat. Psychopharmacology (Berl).

[B6] Rao TS, Correa LD, Reid RT, Lloyd GK (1996). Evaluation of anti-nociceptive effects of neuronal nicotinic acetylcholine receptor (NAChR) ligands in the rat tail-flick assay. Neuropharmacology.

[B7] Damaj MI, Fei-Yin M, Dukat M, Glassco W, Glennon RA, Martin BR (1998). Antinociceptive responses to nicotinic acetylcholine receptor ligands after systemic and intrathecal administration in mice. J Pharmacol Exp Ther.

[B8] Khan IM, Buerkle H, Taylor P, Yaksh TL (1998). Nociceptive and antinociceptive responses to intrathecally administered nicotinic agonists. Neuropharmacology.

[B9] Damaj MI (2000). The involvement of spinal Ca(2+)/calmodulin-protein kinase II in nicotine-induced antinociception in mice. Eur J Pharmacol.

[B10] Ferretti G, Dukat M, Giannella M, Piergentili A, Pigini M, Quaglia W, Damaj MI, Martin BR, Glennon RA (2000). Chain-lengthened and imidazoline analogues of nicotine. Bioorg Med Chem Lett.

[B11] Rashid MH, Ueda H (2002). Neuropathy-specific analgesic action of intrathecal nicotinic agonists and its spinal GABA-mediated mechanism. Brain Res.

[B12] Rashid MH, Furue H, Yoshimura M, Ueda H (2006). Tonic inhibitory role of alpha4beta2 subtype of nicotinic acetylcholine receptors on nociceptive transmission in the spinal cord in mice. Pain.

[B13] Kiyosawa A, Katsurabayashi S, Akaike N, Pang ZP (2001). Nicotine facilitates glycine release in the rat spinal dorsal horn. J Physiol.

[B14] Takeda D, Nakatsuka T, Papke R, Gu JG (2003). Modulation of inhibitory synaptic activity by a non-alpha4beta2, non-alpha7 subtype of nicotinic receptors in the substantia gelatinosa of adult rat spinal cord. Pain.

[B15] Genzen JR, McGehee DS (2005). Nicotinic modulation of GABAergic synaptic transmission in the spinal cord dorsal horn. Brain Res.

[B16] Bellier JP, Kimura H (2007). Acetylcholine synthesis by choline acetyltransferase of a peripheral type as demonstrated in adult rat dorsal root ganglion. J Neurochem.

[B17] Sann H, McCarthy PW, Mader M, Schemann M (1995). Choline acetyltransferase-like immunoreactivity in small diameter neurones of the rat dorsal root ganglion. Neurosci Lett.

[B18] Tata AM, De Stefano ME, Srubek Tomassy G, Vilaro MT, Levey AI, Biagioni S (2004). Subpopulations of rat dorsal root ganglion neurons express active vesicular acetylcholine transporter. J Neurosci Res.

[B19] Bernardini N, Tomassy GS, Tata AM, Augusti-Tocco G, Biagioni S (2004). Detection of basal and potassium-evoked acetylcholine release from embryonic DRG explants. J Neurochem.

[B20] Dixon CE, Bao J, Bergmann JS, Johnson KM (1994). Traumatic brain injury reduces hippocampal high-affinity [3H]choline uptake but not extracellular choline levels in rats. Neurosci Lett.

[B21] Carriedo SG, Yin HZ, Weiss JH (1996). Motor neurons are selectively vulnerable to AMPA/kainate receptor-mediated injury in vitro. J Neurosci.

[B22] Jacobsson G, Piehl F, Meister B (1998). VAMP-1 and VAMP-2 gene expression in rat spinal motoneurones: differential regulation after neuronal injury. Eur J Neurosci.

[B23] Scremin OU, Li MG, Roch M, Booth R, Jenden DJ (2006). Acetylcholine and choline dynamics provide early and late markers of traumatic brain injury. Brain Res.

[B24] Wang W, Salvaterra PM, Loera S, Chiu AY (1997). Brain-derived neurotrophic factor spares choline acetyltransferase mRNA following axotomy of motor neurons in vivo. J Neurosci Res.

[B25] Ichikawa T, Ajiki K, Matsuura J, Misawa H (1997). Localization of two cholinergic markers, choline acetyltransferase and vesicular acetylcholine transporter in the central nervous system of the rat: in situ hybridization histochemistry and immunohistochemistry. J Chem Neuroanat.

[B26] Ichikawa T, Ohsako S, Deguchi T (1991). Production of an antiserum using a fusion protein produced by a cDNA for rat choline acetyltransferase. Neurosci Lett.

[B27] Matsumoto M, Inoue M, Hald A, Xie W, Ueda H (2006). Inhibition of paclitaxel-induced A-fiber hypersensitization by gabapentin. J Pharmacol Exp Ther.

[B28] Lengyel C, Torok T, Varkonyi T, Kempler P, Rudas L (1998). Baroreflex sensitivity and heart-rate variability in insulin-dependent diabetics with polyneuropathy. Lancet.

[B29] Katims JJ (1997). Neuroselective current perception threshold quantitative sensory test. Muscle Nerve.

[B30] Barber RP, Phelps PE, Houser CR, Crawford GD, Salvaterra PM, Vaughn JE (1984). The morphology and distribution of neurons containing choline acetyltransferase in the adult rat spinal cord: an immunocytochemical study. J Comp Neurol.

[B31] Borges LF, Iversen SD (1986). Topography of choline acetyltransferase immunoreactive neurons and fibers in the rat spinal cord. Brain Res.

[B32] Ueda H (1999). In vivo molecular signal transduction of peripheral mechanisms of pain. Jpn J Pharmacol.

[B33] Takeda D, Nakatsuka T, Gu JG, Yoshida M (2007). The activation of nicotinic acetylcholine receptors enhances the inhibitory synaptic transmission in the deep dorsal horn neurons of the adult rat spinal cord. Mol Pain.

[B34] Koga K, Furue H, Rashid MH, Takaki A, Katafuchi T, Yoshimura M (2005). Selective activation of primary afferent fibers evaluated by sine-wave electrical stimulation. Mol Pain.

[B35] Matsumoto M, Inoue M, Hald A, Yamaguchi A, Ueda H (2006). Characterization of three different sensory fibers by use of neonatal capsaicin treatment, spinal antagonism and a novel electrical stimulation-induced paw flexion test. Mol Pain.

[B36] Zhuo M (2007). Neuronal mechanism for neuropathic pain. Mol Pain.

[B37] Sandkuhler J (2007). Understanding LTP in pain pathways. Mol Pain.

[B38] Zhuo M, Gebhart GF (1991). Tonic cholinergic inhibition of spinal mechanical transmission. Pain.

[B39] Naguib M, Yaksh TL (1994). Antinociceptive effects of spinal cholinesterase inhibition and isobolographic analysis of the interaction with mu and alpha 2 receptor systems. Anesthesiology.

[B40] Zimmermann M (1983). Ethical guidelines for investigations of experimental pain in conscious animals. Pain.

[B41] Ueda H, Inoue M (2000). In vivo signal transduction of nociceptive response by kyotorphin (tyrosine-arginine) through Galpha(i)- and inositol trisphosphate-mediated Ca(2+) influx. Mol Pharmacol.

[B42] Hargreaves K, Dubner R, Brown F, Flores C, Joris J (1988). A new and sensitive method for measuring thermal nociception in cutaneous hyperalgesia. Pain.

[B43] Inoue M, Rashid MH, Fujita R, Contos JJ, Chun J, Ueda H (2004). Initiation of neuropathic pain requires lysophosphatidic acid receptor signaling. Nat Med.

[B44] Rashid MH, Inoue M, Kondo S, Kawashima T, Bakoshi S, Ueda H (2003). Novel expression of vanilloid receptor 1 on capsaicin-insensitive fibers accounts for the analgesic effect of capsaicin cream in neuropathic pain. J Pharmacol Exp Ther.

