# Supplementary figures and images for: Evidence for the tonic inhibition of spinal pain by nicotinic cholinergic transmission through primary afferents
Source: Mol Pain. 2007 Dec 19;3:41. doi: 10.1186/1744-8069-3-41 (PMC2234393; doi:10.1186/1744-8069-3-41)

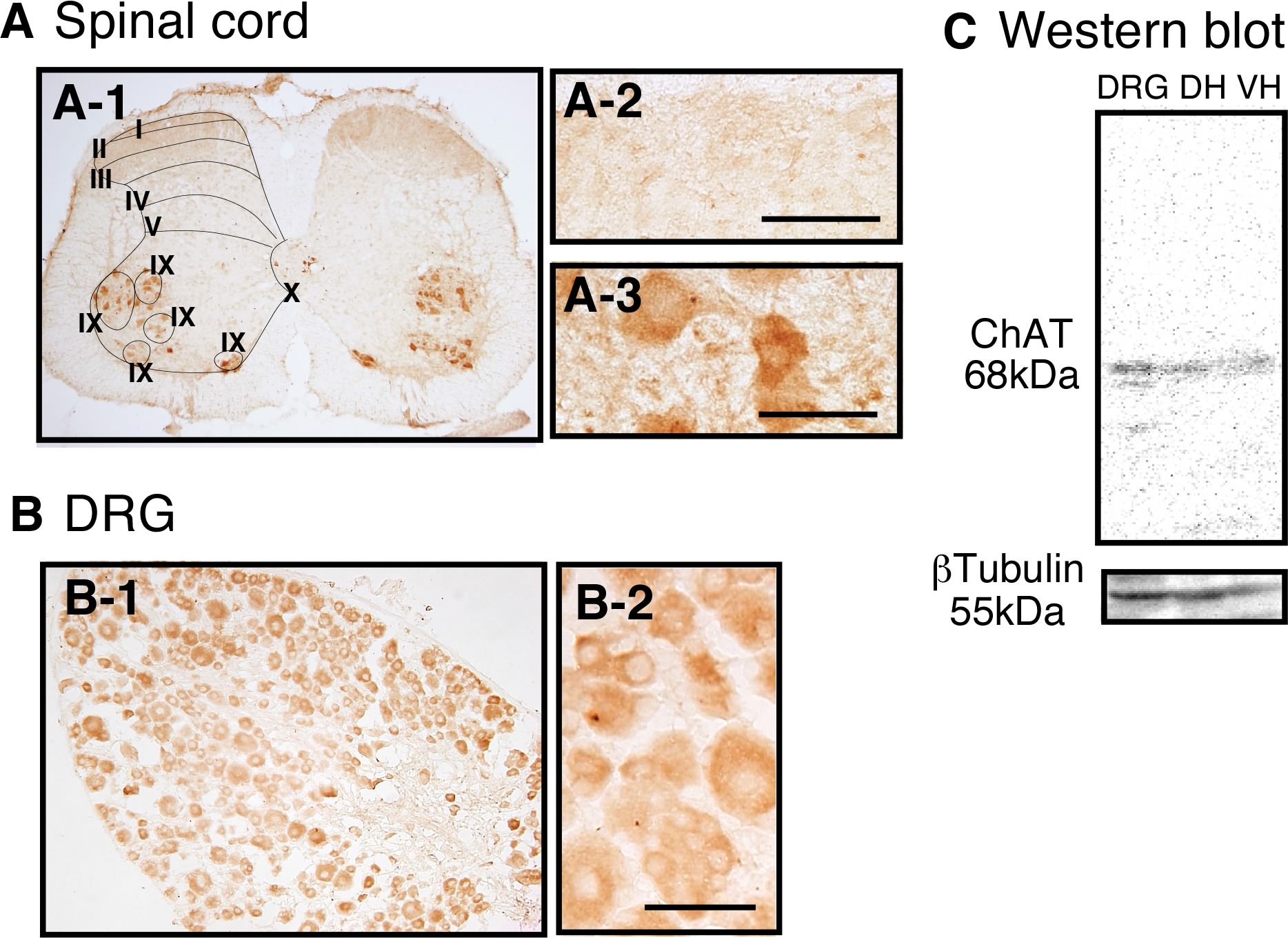

Supplement: Additional file 1 — ChAT-immunoreactivities in the spinal cord and DRG, using a commercially available antibody from a different source (goat anti-ChAT polyclonal antibody, AB144P, Chemicon, CA). (A) ChAT-immunoreactivities in whole spinal cord. (A-1) Representative picture of ChAT-immunohistochemistry in the spinal cord. Many fiber-like structures in the dorsal horn (A-2) and intense ChAT-like signals in the lamina IX region (A-3) were observed. (B) ChAT-immunoreactivities in the DRG. (B-1) Representative pictures of ChAT-immunohistochemistry in the DRG. ChAT signals were found in most cells across the size spectrum (B-2). Scale bar = 50 μm for (A-2, A-3, B-2). (C) Western blot analysis using goat polyclonal antibody indicates one 68-kDa immunoreactive band for ChAT. The 55-kDa immunoreactive band for β-tubulin is also indicated at the bottom. DH: spinal dorsal horn and VH: spinal ventral horn. [file 1744-8069-3-41-S1.jpeg]
